# Guillain-Barré Syndrome Initially Perceived as Unilateral Facial Palsy in Epstein-Barr Virus Infection

**DOI:** 10.7759/cureus.78460

**Published:** 2025-02-03

**Authors:** Keitaro Nagano, Kiyomi Kuba, Masami Osaki

**Affiliations:** 1 Otolaryngology - Head and Neck Surgery, Ageo Central General Hospital, Ageo, JPN

**Keywords:** bilateral facial nerve palsy, epstein-barr virus, facial nerve palsy, guillain-barré syndrome (gbs), incubation period, infectious mononucleosis

## Abstract

We encountered a case of a 21-year-old female presenting with unilateral peripheral facial nerve palsy, initially suspected to be triggered by Epstein-Barr virus (EBV) infection. The patient initially complained of numbness in both lower extremities, progressing to difficulty with mobility by day two, leading to emergency admission. Despite an initial evaluation by a neurologist in the emergency department, Guillain-Barré Syndrome (GBS) was not diagnosed, and she was admitted to internal medicine for further investigation. During hospitalization, she developed infectious mononucleosis and left-sided facial nerve palsy, prompting a referral to our otolaryngology department. Upon further evaluation, subtle weakness on the contralateral side was noted, raising suspicion of bilateral facial nerve palsy. Eventually, the case was diagnosed as bilateral facial nerve palsy associated with GBS. This case underscores the crucial role of otolaryngologists in raising suspicion for GBS when bilateral facial nerve involvement is considered. Recognizing bilateral facial nerve palsy is essential for correctly diagnosing GBS. Since bilateral facial nerve palsy can be subtle, a lack of facial expression may serve as an early diagnostic clue. Additionally, it is important to understand the association between infectious mononucleosis and GBS, as infectious mononucleosis is a common condition. This relationship will be explored in detail.

## Introduction

Guillain-Barré Syndrome (GBS) is a rare but severe neurological disorder characterized by ascending paralysis and involvement of peripheral nerves. While it predominantly affects the lower limbs, it may extend to cranial nerves, including the facial nerves, underscoring the importance of accurate and early diagnosis [1].

The correlation between acute Epstein-Barr virus (EBV) infection and GBS is rare, with a reported incidence of only 0.36% [2]. EBV is also known to cause unilateral facial paralysis [3-7]. Differentiating between unilateral and bilateral facial nerve involvement is crucial for clinical diagnosis, as bilateral involvement often raises concerns for underlying neurological conditions such as GBS. In contrast, unilateral facial palsy in EBV infections can be attributed to Bell’s palsy.

However, diagnosing bilateral facial nerve palsy at an early stage can be challenging, especially when one side of the paralysis is mild.

We encountered a case of a young female presenting with ‘unilateral’ peripheral facial nerve palsy, initially suspected to be caused by acute EBV infection, which was eventually diagnosed as GBS. The delayed diagnosis in such cases risks permanent neurological damage, underscoring the importance of recognizing subtle clinical clues.

Neurological disorders such as GBS are believed to arise due to immunological mechanisms. In GBS, the immune system mistakenly attacks peripheral nerves due to molecular mimicry, where antibodies generated against a pathogen cross-react with nerve tissue, leading to demyelination and neurological dysfunction [8]. While GBS typically follows an antecedent infection [9], EBV-related GBS may exhibit atypical clinical courses, potentially reversing the usual sequence of events.

Through this case, we aim to discuss key diagnostic considerations for bilateral facial nerve paralysis and explore the potential reasons for the reversed onset timing of EBV-induced GBS. We believe that the insights gleaned from this case are pertinent to everyday clinical practice.

## Case presentation

A 21-year-old patient with no known medical history presented to the otolaryngology department with pharyngeal pain which was typical findings of EBV infection along with left facial palsy. Her problems initially started with numbness in both lower limbs. On the second day of the illness, she was unable to get up due to severe fatigue from the bed by herself, and her mother called for emergency medical attention. The neurologist was in denial of GBS, which had been suspected by an emergency physician at first. The patient was admitted to the hospital for further examination. On the day of admission, she first developed a sore throat and liver function abnormalities, and her EBV immunoglobulin M (IgM) was positive, raising suspicion of EBV infection. On the fourth day of hospitalization, additionally, left facial nerve palsy appeared, and the patient was referred to our otolaryngology department for further assessment and management for acute EBV infection and left peripheral facial nerve palsy.

She needed assistance to move from a sitting to a standing position while using a wheelchair and managed to walk unsteadily on her own. In the oral cavity, bilateral palatine tonsils were enlarged and covered with thick white exudate, which was typical of infectious mononucleosis. Blood tests revealed a mild inflammatory reaction, with white blood cell count and C-reactive protein (CRP) levels showing a slight increase, along with mild liver function abnormalities. EBV-IgM was positive, consistent with acute EBV infection. A summary of the blood test results is presented in Table 1.

**Table 1:** Summary of Laboratory Findings in the Patient (Admission Day) Laboratory parameters indicate a mild inflammatory response and mild liver function abnormalities.  EBV VCA-IgM: Epstein-Barr Virus Viral Capsid Antigen Immunoglobulin M, EBV VCA-IgG: Epstein-Barr Virus Viral Capsid Antigen Immunoglobulin G, EBV EBNA-IgG: Epstein-Barr Virus Epstein-Barr Nuclear Antigen Immunoglobulin G, EIA: Enzyme Immunoassay

Test	Value	Reference Range and Unit
White Blood Cell Count (WBC)	6,500/μL	4,000-10,000/μL
C-Reactive Protein (CRP)	1.23 mg/dL	<0.5 mg/dL
Aspartate Aminotransferase (AST)	64 U/L	10-40 U/L
Alanine Aminotransferase (ALT)	66 U/L	10-40 U/L
EBV VCA-IgM	1.6	EIA Index: Negative ≤0.8, Indeterminate 0.9–1.0, Positive ≥1.1
EBV VCA-IgG	0.6	EIA Index: Negative ≤0.8, Indeterminate 0.9–1.0, Positive ≥1.1
EBV EBNA-IgG	0.3	EIA Index: Negative ≤0.8, Indeterminate 0.9–1.0, Positive ≥1.1

The left facial nerve palsy was evaluated as incomplete paralysis, but the overall facial expression was poor and the right side of the face did not look normal. If facial nerve palsy had been evaluated as unilateral, the diagnosis would have been Bell's palsy (idiopathic), and the lower extremity symptoms would have been described as weakness of the lower extremities with severe fatigue. However, based on the patient's history and initial physical examination, GBS was considered more likely, despite the nerve palsy on the right side being subtle.

The neurologist was again requested to conduct a medical examination, considering the possibility of bilateral facial nerve palsy, and the patient was clinically diagnosed with GBS and treatment was started. The patient was treated with intravenous immunoglobulin from the day of diagnosis of GBS. Within two days of starting treatment, the paralysis worsened resulting in a mild respiratory muscle disorder and obvious right facial nerve palsy. Her symptoms subsequently improved significantly and she was discharged with minor paralysis on the 33rd day of hospitalization. She was further evaluated as cured at five months of onset.

## Discussion

While EBV infection can induce Bell's palsy [5,10], it is crucial to actively investigate the presence or absence of lower limb paralysis symptoms and consider the possibility of GBS. A characteristic feature of facial nerve paralysis in GBS is its bilaterality, with paralysis of the lower limbs usually preceding it [11]. In this case, bilateral paralysis was not clear-cut, and the lack of facial expression was considered a clue suggesting the possibility of bilateral facial nerve paralysis. 

In general, GBS can be diagnosed based solely on clinical presentation, particularly the characteristic ascending progression of paralysis, which typically starts in the lower limbs and ascends bilaterally. In 20-60% of GBS cases, facial nerve palsy occurs, predominantly in a bilateral manner [12]. This case aligns with the typical GBS progression, where lower limb paralysis precedes facial nerve involvement, and only a few reported cases have shown facial palsy preceding limb paralysis. Therefore, this case does not deviate from the expected clinical course of GBS.

However, one particular aspect that remains challenging to explain is the sequence of events in this case. Preceding infections in GBS account for 70% of upper respiratory infections and 20% of gastrointestinal infections, with many of these occurring 10-14 days before onset [13]. It raises the question of why paralysis preceded and infectious mononucleosis manifested later in this case. Although there have been case reports of infectious mononucleosis with preceding paralysis as GBS [14], reasons for this sequence have not been mentioned. We speculate that this can be explained by the incubation period of the EB virus. Namely, the EB virus has a significantly longer incubation period of four to six weeks compared to other pathogens causing upper respiratory infections [15]. This prolonged period allows for the immunological establishment of infection, production of antibodies that cross-react with self-antigens, leading to the preceding paralysis symptoms seen in GBS.

## Conclusions

When facial nerve paralysis is suspected in cases of EBV infection, the differential diagnosis should include not only Bell's palsy but also GBS. Early identification of GBS is crucial, as timely intervention is often required to prevent severe complications. Bilateral facial nerve paralysis, though often subtle, is a hallmark feature of GBS. In particular, a lack of facial expression may serve as an early diagnostic clue, especially when accompanied by lower limb paralysis.

This case underscores the importance of interdisciplinary collaboration in diagnosing complex neurological conditions. The delayed onset of infectious mononucleosis symptoms, following the appearance of GBS-related paralysis, highlights the unique pathophysiological relationship between EBV and GBS. The extended incubation period of EBV may provide the immune system sufficient time to produce cross-reactive antibodies, potentially explaining the reversed sequence observed in this case.

By presenting this case, we aim to raise awareness among clinicians about the atypical presentations of GBS associated with EBV. Recognizing subtle signs of bilateral facial nerve involvement and understanding the immunological mechanisms underlying EBV-related GBS are essential for accurate diagnosis and timely treatment.
